# Asian ascendancy: media in the age of globalization

**DOI:** 10.1186/2193-1801-2-646

**Published:** 2013-12-02

**Authors:** Amber Osman, Muhammad Imtiaz Subhani, Syed Akif Hasan

**Affiliations:** Iqra University Research Center-IURC, Defence View, Shaheed-e-Millat Road (Ext.), Karachi, 75500 Pakistan; Iqra University-IU, Defence View, Shaheed-e-Millat Road (Ext.), Karachi, 75500 Pakistan

**Keywords:** Asian Media, Globalization, Media content, Communication

## Abstract

This paper aims to investigate the media industry in Asia, with reference to international standards of media tools, instruments, content, and coverage. We have also explored factors that may further improve Asian media. We have used an empirical approach. Our findings revealed that the media in Asian significantly contribute to expanding cultural knowledge and the exchange of multilateral dialogues. However, they do not look after the interests of minorities or non-dominating communities. Although the media should be a virtual ambassador, they often provoke hostilities within regions. Governments own most media outlets in the developing nations in Asia, and so the media rely on government backing and are subject to restrictions. International and national regulations connected to media freedom or constraints should be explored to protect Asian societies. The practical implications of these negative aspects are that the Asian media does not help the plights of minorities or minimize the fear of war in the region. The universal lesson of brotherhood among humanity for all colors and races should be preached by the media. In this paper, we have concentrated on how Asian media influence cultural expansion, the exchange of multilateral dialogues, the interests of minorities, aggression between nations, and generate income for common citizens.

## Background

Media is the most prominent and strong mode of expression in this age of globalization, and Asia has discovered the power of media to maneuver and manipulate ongoing agendas (Rudolph 1992; Hoselitz 1957). In Asia, the United States, Latin America, and Europe, the top ten activities of teens during the mid-90’s were media associated (Walker 1996). The Asian media setting is different to other parts of the world, although it has been influenced by the west to a certain extent. This difference can be viewed by looking at its performance and operations. The United States has played an important role in learning, creating, and sharing cultural norms. This has been the basis of most media flow (Appadurai 1990; Walker 1996). Appadurai (1990) and Hannerz (1990) described the overlapping of Asian consumer culture with the United States, which is facilitated by the US media. From snack food to fashion to furniture, consumer culture schemes (lifestyle, aesthetics, and language themes) are channeled from the United States to other parts of the world through mediascapes. Media allows people to communicate through different sources. In both media law and practice, Asian countries are similar to international standards. Djankov et al. (2003) found that government control of the media (related to less liberty of the Press) is greater in countries with autocratic political establishments. The Asian media is open and democratic (for the people and by the people). Public interest and government drives the Asian media for the betterment of the community (Djankov et al. 2003).

In the recent years, Asian media outlets have tried to overcome and challenge their western opponents who control the world media market. A renowned Asian news agency’s editor-in-chief stated that the information of the world flows from developed countries to developing countries. Another New York Times columnist said that the magic power of globalization has been proven by famous entrepreneurs like Bill Gates (Harrison 2012).

One seventh of the world’s population is from Asia and developed countries, but they control two thirds of the total information flow. In addition, Asia has tried to be fairer, objective and provide more comprehensive coverage than western media. Asian media agencies, particularly from the developing nations, are now actively participating in world events and collaborate more when reporting regional news. There is no longer a monopoly for reporting important regional news and events in Asia, as media outlets are now sharing, editing, and exchanging information and reports to provide better media content (Chan J, Suen W 2008).

Asian media are researching new technologies to send news over the internet and cellular phones. Asian media have been developing and improving since the 1960s, fighting against unjust control by the western developed nations, and are now becoming globally dominant.

Disparities of the media content and information flow between developed and developing countries has not improved in decades, because of the western media’s misapprehensions and biased opinions regarding developing and Asian countries. Asia has learnt to invest in its own and relocate its resources to encourage better communication and gain power. Recent developments include communication meetings and groups where, for example, countries like China, Japan, Korea, and Sri Lanka have signed agreements to speak louder than the rest of the world (Xinhua 2007)*.*

Time and place are always important, and the same is true for media. Cultural barriers have created differences in media content. From the organization perspective, there are hindrances to entering into foreign markets because of the demands of culturally reliant spectators. Increases in the global flows of commodities and people mean that we have entered into ‘imagined worlds’, where social connections are made without borders, flags, or national interest. International audiences lack familiarity with signs and symbols. Language barriers are another difference, which vary from country to country. Media content is translated into the language of local audiences, but language barriers still influence the success of the media content (Rohn 2010).

Media represents the modern era; it accepts and broadcasts the positive and negative events that are happening around us. It acts as a platform to reveal and address various issues from different perspectives. However, it also generates positive appearances and expressions, and reflects on the history ingrained in societies. This paper is an empirical attempt to highlight the role of media in the age of globalization in Asia.

Challenges in Asian media are related to cultural politics. The first challenge is the competition to produce culture, and arguments over its meaning. Modern society seeks individual identity and worth, which may be visible through media, and propagates the circulation of power, wealth, and status. The second challenge is that media’s key interest is to generate the public’s culture at the same time as encouraging socio-economic gain (Rudolph 1992). It is a challenge and an opportunity that media can reveal and hide the realities of society. The realism, symbolism, idealism, classism, and romanticism of society can be allegorically or non-allegorically reported and addressed by the media. The media expands culture through globalization and the international exchange of dialogues, but sometimes by reporting opposing views regarding different sovereigns they can increase hostilities between nations.

Media substantially contributes to economy and production. As studied by the World Bank, Locksley (2008) identified that the principal level, which is *transparency and multitude,* supports and nurtures media as a whole and provides good power/governance for political and economic markets.

It is a top-down and bottom-up approach because the media reaches out to the powerful and powerless, to the rich and the poor, to safeguard the interest of the particular, create a sense of responsiveness, and make government and policy makers accountable for the environment’s safety and protection.

The expense of advertising on a global scale is because of the media’s ability to control and sway *behavior*. A large amount of money is spent to persuade and bring change in individuals, groups, and organizations worldwide. The effectiveness of the media in encouraging transformations or changing opinions varies (Bryant and Zillmann 2002; Hornik 2002).

### Infrastructure and platform

The infrastructure and platforms used by the media have been developed because they move information from one station to another. The appropriate use of devices generates investments and a shift in economy. The use of new broadband digital media has changed global communication and increased revenues (Dagron 2001).

The next generation broadband networks can deliver TV programs, films, music, games, radio, and publishing in synergy. Competition exists because the operators of infrastructure are always generating better results through quality, choice, innovation, and price (Locksley 2008). In Asia, countries like China have advanced media infrastructures and platforms, and have undergone a digital switchover to raise their economy. Some developing countries still require a change to uplift their economy and align with infrastructure changes elsewhere. In addition, mobile networks are an important part of media infrastructure and are very popular with the audience. Mobile networks are an innovation that have allowed communication between the media and public, for example it is now possible for an individual to use SMS to vote, give an opinion, or to share news happening around the corner (Locksley 2008, p. 10).

### Economic

The media industry generates significant income and employs many people, which helps a nation’s development and reduces poverty (Locksley 2008, p. 11).

The media industry is large and can be divided into pre-production, production, postproduction, sharing (platforms), and archiving (storage for reuse). All of these sub-divisions are globally connected because media activities take place at different locations. Around the world, different cultural assets and resources are found, captured, and shared by media in a creative way, which adds to economic worth and development. Media organizations have employed people from diverse cultures. In developing countries, cultural diversity should be encouraged to expand media operations to create more job opportunities, improve technology, create value, export development, and maximize wealth (Sagnia 2005).

### Trade

Trade in media relates to its content production. Economically, the media trade is not straightforward. Economy of scale and scope helps media to saturate markets internationally. The Asian market has gradually extended its distribution channels to recoup expenditures (Locksley 2008).

Asian media’s other challenge is to act in the interest of the minorities of the continent, because it is worthwhile and an opportunity. Market and media are symbiotic to each other. In Pakistan, the media has complete freedom of expression and communication since the Musharraf era. The media can be used to gauge and defend the country to a certain extent (Musharraf 2006).

Media has many different trends, most have a history, but some only exist since the digital age and some are new. As these trends continue, they will influence the contribution of the media to society. Media trends in Asian countries are only recently being applied, especially in countries like China, Singapore, and Japan. The irrelevance, packaging, venture capital, cascade, regulatory, abundance, globalization, fluidity, and concentration factors (media production features) are carefully practiced in this industry (Locksley 1988).

Social media (i.e., twitter, facebook) have become more popular, and now there are millions of users around the globe (Yu et al. 2011). Social network use has given more opportunities to connect, share, and communicate with a continual stream of news, videos, images, and discussions. It has had a massive impact on online computing systems (Yu et al. 2011). Various authors have studied social networks such as Orkut, Flickr, myspace, and youtube, investigating their international distributions (Mislove et al. 2007).

Asian countries like Sri Lanka, Bangladesh, Pakistan and India are adopting these new and innovative technologies but at a slower rate than other Asian Countries (UN News Center 2011; Ismail 2013). The underlying factors behind this slower uptake are recession, inflation, and poverty. Media is helping these societies to rise as nations, adopt innovation, and experience colorful lives. The media are dominant in Asia through dramas, movies, morning shows, live talks, radio, historic moments, health knowledge, and education. The establishments of different industries are shown to the viewers for judgment (Global Forum on Media Development 2007).

Media has no religion. It is a communication spree that is connected, global and accessible. There have been many international collaborations and decentralized networks that use different mediums, such as CNBC. The BBC also has integrated news channels in different countries, including Asian countries. Voice of America has radio channels connected to Pakistan and many other Asian countries. Famous American and European celebrities are used as international brand ambassadors who create a favorable association in Asia through media channels, print, advertisements, etc. (European Commission 2007). Cultural diversity in media can maintain positive international relationships, discouraging aggression and encouraging understanding of different beliefs and values. Most importantly, in the future the media must mitigate the dominance of a single social group in the name of autonomy, freedom, and international free-trade of communication (Ambirajan 2000). The actions and behaviors of the media should not be antagonistic, but they should penetrate cultural aspects of this modern globalized and progressive world to achieve undeviating economic dominance (Ambirajan 2000). The world is often in turmoil owing to aggressive behaviors and power struggles. Mankind can be destructive, using weapons to rule the world. Various conflicts are being caused by religion, economics, politics, science, social problems and technologies. Different communities of the world and different individuals do not think and act in the same manner, and their differences often result in warfare (FES Pakistan 2012). The Asian media tries to fix national and international issues, but to get the best of globalization and freedom they must emphasize sub-cultures and control centralized networks. In short, the media in Asia must continue to function with a mixture of heterogeneity and homogeneity (Rudolph 1992; FES Pakistan 2012).

Consumer preferences are extremely diverse in today’s global village and technological society. Ethics is an eminent issue, in particular wisdom, self-discipline and judgment (Ainslie 1992; Casson 1998; Charlton 1988). Media ethics have been debated since the 1960s. Media coverage can change the behavior of consumers, so other organizations are interested in taking advantage of these influences (Kim and Choi 2007).

The latest debates in media education have shown how important it is to understand the significant potential for the media to impart knowledge and information. Work on media literacy has been ongoing since the 1990s and the media use two models: protectionism and preparation (Buckingham 2003; Hobbs 1998; Von Feilitzen 2000, 2004). These studies consider the democratic media and youth working in the media industry. Media production is considered independent and young talent is considered to have a sense of global critique. This helps to develop a thorough vision of media literacy. The media shows multiple views of life that can damage youth, but they cannot be isolated from it. Damages include a detrimental effect on language and youth expression, and leading youth away from religious values and rituals. For example, Indian soaps have a bad impact on children’s language and expressions and are distancing them from religious and cultural norms (Mukhtar 2013). Buckingham (2003) and Hobbs (1998) believe that this damage can be mitigated through media-aided mentoring by educators. Educators should encourage youth by using the media (Poyntz 2006).

## Methods

### Research questions

To investigate Asian media in this age of globalization, we developed the following research questions.

1. Do the media expand Asian culture?
2. Do the media facilitate the exchange of multilateral dialogues in Asia?
3. Do the media look after the interests of minorities in Asia?
4. Do the media ease the risk of warfare between Asian nations?
5. Do the media generate income in Asia?
6. Which production features (packaging, venture capital, regulatory, abundance, globalization, fluidity, concentration, and irrelevance factors) are being practiced the most in the Asian media?

### Description of data and sampling

This paper is investigating a very broad subject area. We have used a survey to collect the relevant data from a large sample of the population (McIntyre 1999, p. 74). To gauge the role of the media in Asia, we consulted top, senior, and middle level media professionals and audiences from different continents of the world. We used a sample of 10,000 respondents (5000 media professionals and 5000 media viewers from all over the world). There was a 66% response rate. Data were gathered from Asia (1500 respondents), Europe (1400 respondents), Australia (1400 respondents), Africa (1400 respondents), North America (1425 respondents), South America (1425 respondents), and the Middle East (1450 respondents). We used a non-restricted non probability sampling (convenience sampling) method to collect information regarding the Asian media. Emails were used for respondents from Pakistan, India, Afghanistan, Iran, China, Japan, and Singapore, and telephone and personal visits were used within Pakistan. We took care to avoid non response items and to keep costs minimal. These Asian countries were considered because of their common geography. The questions were set according to ethical research standards, and no objectionable or personal questions were asked from the respondents. A set of questionnaires were sent to the research ethics committee of Iqra University for review and discussion, and a majority of members of the committee agreed to a modified questionnaire. We designed closed-ended questions that used the Likert scale (a numerical range from strongly agree (5) to strongly disagree (1)) to gauge the responses of the respondents (Salant and Dillman 1994, p. 96–97). We tested the reliability of the questionnaire using Cronbach’s alpha. The reliability coefficient was 0.72, which demonstrates that the questionnaire is reliable. We validated the data using cross variable validation and found no violations in the collected data.

We are interested in beliefs, attitudes, and behaviors in the Asian media and the interrelated variables discussed in the previous section. It is always difficult to comprehend subjective responses and opinions and determine the adequacy and coherency of the answers, so we designed a sequence of connected questions (Salant and Dillman 1994, p. 90).

The sample size was 10,000, which included the pilot testing of the questionnaire and did not include incomplete responses. We randomly chose countries from every continent based on peer contacts, family and friends. Iqra University (the parent organization of Iqra University Research Center) helped our team achieve the desired results, providing sufficient financial and technical support.

When the survey was completed, we coded and processed the data. Because the data were categorical in nature, we used optimal scaling/categorical regression to determine the relationships between the questionnaire answers and our research questions. We used the median score to check the ranks of the production features used in Asian media.

## Results and discussion

Table 1 highlights some of our important findings. As can been seen from the results, Asian media in this time of globalization significantly contribute to cultural expansions in the region (beta = 1.562, t = 27.142). The results also show that the Asian media significantly aid multilateral dialogues among the Asian nations (beta = 10.687; t = 15.410). We also found that the Asian media as a whole are not working in the interests of minorities (beta = -0.040; t = -0.102). The Asian media provoke disagreements in the region instead mitigating them (beta = -0.081 at t = -16.154). However, they are generating revenue in the region (beta = 0.771 at t = 8.879).

**Table 1:** **Categorical regressions**

Dependent		CE	FEMD	LIM	RHNN	IG
Variables						
Independent						
Variable						
MAG	Beta	1.562	10.687	-0.040	-0.081	0.771
	(T-Stats)	(27.142)	(15.410)	(-0.102)	(-16.154)	(8.879)
	R-Squared	0.700	0.424	0.503	0.304	0.200
	(F-Stats)	(421.031)	(3.844)	(204.010)	(3.916)	(1.364)
	In Asia as a whole, the media significantly contribute to cultural expansion and the exchange of multilateral dialogues. However, they are not looking after the interests of minorities or non-dominating communities.					

Note:

CE= Cultural expansion.

FEMD= Facilitating exchange of multilateral dialogues.

LIM= Looking after the interests of minorities.

RHNN= Reducing hostilities between neighboring nations.

IG= Income Generation.

MAG= Media in the age of Globalization.

Table 2 displays our findings on the contributions of Asian media in different regions. The Pakistani media is expanding culture in the region, facilitating multilateral dialogues, looking after the interest of Pakistani minorities, and fostering good relationships with neighboring nations (note the significant beta coefficients and their respective t-statistics, which are all greater than 1.5). However, they are not contributing to raising income in its region (the beta coefficient and t-statistics are not significant, i.e., t-stats < 1.5).

**Table 2:** **Categorical Regressions by Region**

Dependent			CE	FEMD	LIM	RHNN	IG
Variables							
Independent							
Variable							
MAG	PAKISTAN	Beta	0.424	0.373	0.260	0.210	0.183
		(T-Stats)	(1.731)	(1.610)	(2.458)	(1.551)	(1.023)
		R-Squared	0.451	0.411	0.527	0.375	0.212
		(F-Stats)	(4.991)	(3.927)	(22.071)	(3.888)	(3.008)
		In Pakistan, the media contribute significantly and positively to cultural expansion, the exchange of multilateral dialogues, the interests of minorities, and the reduction of hostilities between neighboring nations (t-stats > 1.5 and positive beta coefficients). However, they do not necessarily contribute to income generation (t-stats = 1.023).					
	INDIA	Beta	0.738	0.402	0.153	0.102	0.312
		(T-Stats)	(2.563)	(2.105)	(1.411)	(1.005)	(8.740)
		R-Squared	0.661	0.327	0.227	0.319	0.572
		(F-Stats)	(103.017)	(11.582)	(3.939)	(3.873)	(11.011)
		In India, the media contribute significantly and positively to cultural expansion, the exchange of multilateral dialogues, and income generation (t-stats > 1.5 and positive beta coefficients). However, they do not contribute to the interests of minorities or reduce hostilities between neighboring nations (t-stats < 1.5).					
	AFGHANISTAN	Beta	0.019	0.101	0.062	0.004	0.013
		(T-Stats)	(1.002)	(0.519)	(1.031)	(0.408)	0.111
		R-Squared	0.229	0.002	0..007	0.201	0.061
		(F-Stats)	(1.384)	(2.826)	(1.732)	(2.410)	(1.983)
		In Afghanistan, the media do not significantly contribute to any of these factors (t-stats < 1.5).					
	IRAN	Beta	0.327	0.219	0.116	0.010	0.071
		(T-Stats)	(1.501)	(0.729)	(1.171)	(0.003)	0.020
		R-Squared	0.106	0.037	0.373	0.101	0.201
		(F-Stats)	(3.871))	(2.613)	(3.951)	(1.111)	(1.389)
		In Iran, the media do not contribute significantly and positively to cultural expansion (t-stats > 1.5), but they do contribute to the other factors studied (t-stats < 1.5).					
	CHINA	Beta	0.799	1.794	0.251	0.177	1.413
		(T-Stats)	(14.032)	(9.483)	7.004	(13.063)	(2.653)
		R-Squared	0.543	0.672	0.419	0.090	0.384
		(F-Stats)	(76.001)	(100.052)	(22.732)	(5.007)	(3.979)
		The Chinese media contribute significantly and positively to cultural expansion, the exchange of multilateral dialogues, the interests of minorities, reducing hostilities between neighboring nations, and income generation income (t-stats > 1.5 and positive beta coefficients).					
	JAPAN	Beta	0.507	0.331	0.165	0.132	1.051
		(T-Stats)	(1.993)	(1.809)	(7.731)	(1.771)	(2.002)
		R-Squared	0.630	0.013	0.400	0.234	0.313
		(F-Stats)	(8.451)	(4.708)	(20.001)	(4.641)	(3.872)
		The Japanese media contribute significantly and positively to cultural expansion, the exchange of multilateral dialogues, the interests of minorities, reducing hostilities between neighboring nations, and income generation income (t-stats > 1.5 and positive beta coefficients).					
	SINGAPORE	Beta	0.231	0.102	0.150	0.165	1.001
		(T-Stats)	(1.004)	(1.841)	(0.002)	(2.030)	(1.009)
		R-Squared	0.076	0.010	0.001	0.201	0.006
		(F-Stats)	(3.003)	(4.009)	(1.023)	(4.372)	(1.077)
		In Singapore, the media do contribute significantly and positively to the exchange of multilateral dialogues and reduce hostilities with neighboring nations (t-stats > 1.5 and positive beta coefficient). However, they do not significantly contribute to cultural expansion, the interests of minorities, or income generation (t-stats < 1.5).					

Note:

CE= Cultural expansion.

FEMD= Facilitating exchange of multilateral dialogues.

LIM= Looking after the interests of minorities.

RHNN= Reducing hostilities between neighboring nations.

IG= Income Generation.

MAG= Media in the age of Globalization.

Table 2 also highlights the contribution of the Indian media. The data revealed that the Indian media contribute significantly and positively to expanding culture, facilitating multilateral dialogues, and income generation (significant beta coefficients and their respective t-statistics, i.e., all t-statistics are greater than 1.5). However, they do not look after the interest of minorities or ease aggression with neighboring nations (insignificant beta coefficient and t-statistics, i.e., t-stats < 1.5).

The Afghani media do not significantly contribute to any of the areas of our investigation (all beta coefficients and their respective t-statistics are insignificant, i.e., all t-statistics are less than 1.5).

The Iranian media only contribute to the cultural expansion of the (significant beta coefficient and t-stats > 1.5). They fail to facilitate the exchange of multilateral dialogues, look after the interests of minorities, ease aggression with neighboring nations, or generate revenue (t-stats < 1.5).

The Chinese and Japanese media contribute significantly and positively to all of the outlined areas, i.e., they expand culture, facilitate the exchange of multilateral dialogues, look after the interests of minorities, ease aggression with neighboring nations, and generate income (positive beta coefficients and t-stats > 1.5).

In contrast to the Chinese and Japanese media, the Singaporean media only contribute significantly and positively to facilitating the exchange of multilateral dialogues, and easing aggression with neighboring nations (positive beta coefficients and t-stats > 1.5).

The findings displayed in Table 3 uncover that in this industry, the most commonly used media production factor is the packaging factor, as the median scores for this factor are greater than median scores of all other factors for all outlined nations.

**Table 3:** **Median scores for practicing factors in Asian media**

Countries	Pakistan		India		Afghanistan		Iran		China		Japan		Singapore	
Media Production Factors	S	R	S	R	S	R	S	R	S	R	S	R	S	R
Packaging Factor	4.0	1	4.4	1	4.5	1	3.9	1	4.6	1	4.5	1	4.4	1
Venture Capital Factor	3.9	2	4.0	2					4.3	3	3.9	3	3.9	3
Regulatory Factor	3.7	3					3.5	2						
Abundance Factor					3.6	2								
Globalization Factor			3.9	3					4.4	2	4.3	2		
Fluidity Factor					3.3	3								
Concentration Factor													4.0	2
Irrelevance Factor							3.7	3						

Note:

S= Scores.

R= Ranks.

The packaging factor is being practiced the most in Asia (Pakistan, India, Afghanistan, Iran, China, Japan, and Singapore) as this has the greatest median scores in all regions.

## Conclusions

We conclude that the media in Asia as a whole significantly contribute to cultural expansion and the exchange of multilateral dialogues. However, they do not look after the interests of minorities, although there have been minor improvements in relation to religious minorities. It should be noted that the media are virtual ambassadors who should always attempt to mitigate hostilities between nations, but it can been seen from our results that they can often provoke aggressions between territories. Asia has become an emerging economic source of power and this economic advancement will determine military modernization and any form of war intervention. The findings further reveal that, in Asian media the packaging factor production feature is the most practiced feature (see Table 3).

The findings of this paper unfortunately highlight that the media has failed to look after the rights and interests of minorities. This was confirmed during the massacre at Burma this year 2012, which was not reported by any of the Asian media outlets. It is also disheartening that the findings of this paper show that in this region the media provokes hostilities instead of mitigating the chance of wars. This has been seen in the case of drone attacks on tribal areas in Pakistan (although primarily used to attack and destroy militants, war crimes have been committed because of damage to innocent civilians), and the threat of US attacks on Iran and North Korea because of negative propaganda from the Indian media (Mazhar & Goraya 2011). In contrast to these issues, the media has promoted cultural expansions and multilateral dialogues among the nations of the region.

Media trends are nurturing two-way communication for information exchange. This also creates job opportunities in desirable locations (Landry 2000). We also noted that communication is difficult in rural areas, making it difficult to influence and help some communities. Recent developments have targeted these areas, particularly because of floods and natural disasters. The media reported on flood and earthquake victims in Pakistan and Japan to encourage the rest of the world to send aid. Many more similar incidents are being reported on to influence governments and policy makers.

In Asian countries that are developing, poor, more autocratic, or less educated governments own the media because they use public choice theory to handle political and economic freedom. China, Japan, Korea, India, Sri Lanka and many other countries control information communication technology (ICT) by enforcing policies and deploying certain projects (Shiming 2008).

Asian nations have initiated collaborations with media to foster an extremely strong media industry in the near future. These include the following.

- Maintaining cultural resources and encouraging creativity.
- Encouraging small/medium enterprises and controlling micro-finances.
- Enforcing laws and copyrights.
- Overall protection and backing of media artists via institutions, both monetary and non-monetary.
- Restoring and retaining cultural legacy.
- Stimulating digital industry growth and understanding.
- Expanding into domestic and foreign markets.
- Encouraging tourism.
- Imparting of education, training, and skills.
- Offering investment options and tax exemptions.

The Asian media should continue developing this industry by incorporating true value using the following outline.

- Freedom of speech and expression.
- Guiding principles and rules.
- Capacity enlargement.
- High standards of professionalism and moral principles.
- Content.

Asian governments are trying to maintain stability and revenue returns in media. It is now evident that the Asian media expanded very quickly. Politics has a major role in the media. Commercialization of media has made it more powerful, public, and profitable. In this race, it is very important to concretely define what is right and what is wrong, and decide whether media content has a positive outcome. Media is the only platform and channel to unite people from different castes, creeds, and races during tragedies and during celebrations. The policy makers, stakeholders, and new entrants into this domain should recognize the basic purpose of the media, which is to explore, visualize, and share information that may improve the world.
